# Sustainable agriculture

**DOI:** 10.1186/s12870-023-04626-9

**Published:** 2023-11-25

**Authors:** Subhojit Datta, Islam Hamim, Durgesh Kumar Jaiswal, Rungroch Sungthong

**Affiliations:** 1 Biotechnology Unit, Division of Crop Improvement, ICAR-Central Research Institute for Jute and Allied Fibres, Barrackpore, Kolkata, West Bengal 700121 India; 2 Department of Plant Pathology, Bangladesh Agricultural University, Mymensingh, 2202 Bangladesh; 3 Functional Biodiversity, Institute of Evolutionary Ecology and Conservation Genomics, University of Ulm, Ulm, 89081 Germany; 4 Department of Biotechnology, Graphic Era(Deemed to be University), Dehradun, Uttarakhand 248002 India; 5 Department of Biochemistry and Microbiology, Faculty of Pharmaceutical Sciences, Chulalongkorn University, Bangkok, 10330 Thailand

## Abstract

Developing sustainable agricultural practices is currently becoming an increasingly relevant challenge. As the worldwide population rises and climate change affects agriculture globally, new and sustainable approaches must be adopted to ensure food security. In this editorial, we invite contributions to a *BMC Plant Biology* collection on ‘Sustainable agriculture,’ covering research on the environmental and socioeconomic factors that affect sustainable agricultural practices and their management.

## Crop improvement in the era of multi-omics to face the challenges of climate change and rising world population

In line with the United Nations Sustainable Development Goal 2 (SDG2), food security represents a major and global challenge, as the world’s population increasingly grows. In addition, agricultural production is globally threatened by climate change. Over the years, the development of crops with higher yields and resilience to biotic and abiotic stressors has become a key strategy for enhancing agricultural production. Recent developments in omics technologies (e.g., genomics, transcriptomics, proteomics, and metabolomics) allowed us to better comprehend crops’ metadata and earn benefits from such data. Omics-based technologies led to the identification of numerous drought-tolerance genes and over expression of these genes resulted in the development of drought-tolerant maize lines, which have been shown to have a higher grain yield under water stress conditions [1]. Elucidation of the complex transcriptional rearrangements upon Tomato Leaf Curl New Delhi Virus (ToLCNDV) infection in melon is another example of how omics technologies can pave the way for gene discovery and characterization [2]. The above two examples from a cereal crop and a horticultural crop demonstrate the power and relevance of omics-led discoveries in understanding complex genetic networks in plants and developing novel technologies for sustainable agriculture. During recent years, we have seen that multi-omics approaches have rapidly developed, offering promising tools for crop optimization to ensure food security.

## Sustainable management of plant pests and diseases: from molecular perceptions to field applications

Due to the constant exposure and interactions of plants with their pests and pathogens, innovative fundamental and translational studies are required to develop efficient and sustainable pest and disease control strategies. These research areas encompass the molecular mechanisms underlying the plant-pathogen and plant-pest interactions, as well as the ecology and epidemiology of such interactions in the environment. The discovery of previously unidentified pathogens and pests, the identification of novel genes and molecular mechanisms underlying plant-pathogen and plant-pest interactions, and the development of pest- and disease-resistant plants are all examples of significant advances in molecular studies in the field of plant protection and management [3]. Emerging molecular tools such as long-read sequencing technologies are transforming the detection and genomic characterization of plants and their pathogens and pests [3], as well as how we understand and characterize their interactions [3], disease and pest management strategies [4, 5], and the development of new plant varieties or cultivars [3]. Molecular research on plants, pathogens, and pests in the ecosystem has led to the development of genetically modified plants with resistance to pests and diseases, some of which persist for a long time. Due to concerns about possible adverse effects on the environment and human health, the use of genetically modified crops is still controversial. However, sustainable alternatives are potentially available for pest and disease management in the field [4, 5], for instance, the use of biological control agents like antagonistic microorganisms or naturalenemies of pests. For example, several mycoviruses are reported as biocontrol agents against plant diseases and insects [5]. Although these methodsare advantageous for the environment and human health, their efficacy may be constrained by factors like the environment, the pest and pathogen populations, and the accessibility of appropriate biological control agents [5]. In addition, the use of cultural practices such as crop rotation, intercropping, and resistant plant varieties, represents valuable approaches for the long-term control of pests and diseases [4]. A continuous investigation into the molecular, ecological, and epidemiological aspects of plant interactions with pathogens and pests in the field, however, is required for the application of integrated approaches and the development of sustainable methods for managing plant pests and diseases.

## Microbial inoculants: an alternative approach to promote sustainable agriculture

Sustainable food production can only be accomplished if agricultural productivity increases by 70%, despite the harsher weather conditions, and if environmental quality is maintained (e.g. by reducingthe use of agrochemicals) [4]. Many national and international organizations have encouraged the development of sustainable alternatives that use existing natural resources to achieve food security under climate change and limited land resources [4]. Therefore, in the burgeoning field of soil microbiology, microbial inoculants have emerged as a viable technique in sustainable agriculture systems. Microbial inoculants can enhance crops yield by increasing plant nutrient availability, and reducing abiotic stress (e.g. drought, heat, cold and salinity) [4, 5] and biotic stress (e.g. diseases and pests) [6, 7]. Nevertheless, the range of effects of microbial inoculants on crops production appears to change in relation to their different mechanisms of action, as well as the crop types and cultivation conditions used [6]. A quantitative assessment of the different effects of microbial inoculants still needs to be achieved. In this context, there is the necessity to (i) investigate the impact of microbial inoculants on crops growth, yield, and nutritional quality, (ii) identify the mechanisms by which microbial inoculants enhance crops performance, (iii) understand why the effect of microbial inoculants varies between crop types and cultivation conditions, and (iv) achieve their large-scale and efficient use. Therefore, further research is required to understand how beneficial microbes and soil/plant microbiomes contribute to agronomic practices under different climate conditions. This will enable to explore and identify the most suitable microbial inoculants for different agricultural contexts and develop synergistic tools or strategies to achieve sustainable crop production.

## Accessible crop production systems: how to make food under limited resource conditions

Some of the critical consequences of global warming and rising world population include drought, land deterioration, and farming area invasion due to urban expansion [8, 9]. These issues directly affect modern agriculture and impede crop productivity, as highlighted in the UN Sustainable Development Goal 15 (SDG15). Some innovations in sustainable agricultural practices, like vertical farming, hydroponics or aquaponics, have been developed to reduce land use, optimize irrigation, and maximize nutrient recirculation [8–10]. While growing crops by employing alternative approaches (e.g. vertically) can improve space usage, it may also require considerable energetic costs (e.g. water and nutrient supply). The use of solar energy as an alternative power supply in such crop production systems has been assessed and shown to reduce energy consumption [8]. As nutrient recycling is central to ‘soilless cultivations,’ various strategies have been studied and implemented for the supplying and monitoring of nutrients, such as nanoparticles-enhanced nutrient supply, beneficial plant-microbe interactions, the use of real-time sensors to track nutrient levels, or the integration of data science with smart agricultural practices [9]. Aquaponics, which is a food production system that couples aquaculture with hydroponics, is another soilless approach employing crop plants as biofilters of waste products or nutrients (e.g. from fish farming) that are ultimately utilized for their growth [10]. Although all these attempts are promising strategies to address global food security concerns, knowledge gaps remain to be fulfilled and existing technologies must be improved to become applicable in sustainable agriculture.

## Conclusions

The development of sustainable agricultural practicesis critical to meet the global increasing need for food, while reducing the negative consequences of agricultural activities on the environment. To promote research and innovation, knowledge exchange and community engagement in sustainable practices for global agriculture, *BMC Plant Biology* welcomes submissions of research articles to the collection ‘Sustainable agriculture’. The collection focuses on research areas such as crop yield enhancement, resource management and ecosystem services, sustainable control of plant pests and diseases, including diagnosis, characterization and management, while also encouraging the development of sustainable methods that address the problems of soil erosion, climate change, and biodiversity loss. This collection provides a platform to promote sustainable agriculture, helping researchers, decision-makers, and farmers who are pursuing agricultural practices for a sustainable future.

## Data Availability

Not Applicable.
